# A comparative assessment of generalized anxiety, conduct and peer relationship problems among AIDS and other orphaned children in India

**DOI:** 10.1186/s12888-016-1042-z

**Published:** 2016-09-21

**Authors:** Prem Kumar SG, Anil Kumar G, Ramgopal SP, Venkata Srinivas V, Rakhi Dandona

**Affiliations:** Public Health Foundation of India, Plot 47, Sector 44, Gurgaon, 122 002 India

**Keywords:** AIDS, Generalized anxiety, Children, Conduct problem, HIV, India, Mental health, Orphans, Peer relationship problem

## Abstract

**Background:**

Data on mental health among orphaned children in India are scanty. We compared the generalized anxiety, conduct and peer relationship problems and their associated risk factors among children orphaned by HIV/AIDS and those due to other reasons in the Indian city of Hyderabad.

**Methods:**

Four hundred orphaned children aged 12 to 16 years residing in orphanages in Hyderabad were sampled, half being AIDS orphans (COA) and the rest orphaned due to other reasons (COO). Interviews were done using standardized scales to assess generalized anxiety, conduct and peer relationship problems. A score >8, >4, and >5 was considered as indicator of generalized anxiety, conduct problem and peer relationship problem, respectively. Variations in the intensity of these three conditions due to possible factors including co-existing depression were assessed using multiple classification analysis (MCA).

**Results:**

A total of 396 (99.3 %) orphans participated of whom 199 (50.3 %) were COA. The mean generalized anxiety, conduct and peer relationship problem scores were 11.1 (SD 5.2), 3.8 (SD 2.5) and 3.8 (SD 2.5) for COA; and 7.6 (SD 4), 2.6 (SD 2) and 2.3 (SD 1.8) for COO, respectively. Among COA, the prevalence of generalized anxiety score of >8 was 74.4 % (95 % CI 67.8–80.0 %), of conduct problem score of >4 was 33.2 % (95 % CI 26.9–40.1 %), and of peer relationship problem score of >5 was 27.6 %, (95 % CI 21.8–34.3 %), with these being significantly lower in COO. In MCA, a higher mean depression score had the highest effect on the intensity of generalized anxiety, conduct and peer relationship problem (Beta 0.477; 0.379 and 0.453 respectively); being COA and a girl had the most impact on generalized anxiety (0.100 and 0.115, respectively).

**Conclusions:**

A significantly high proportion of AIDS orphans deal with generalized anxiety, conduct and peer relationship problem as compared with other orphans highlighting the need to address the poor mental health of orphans in India.

## Background

With the recent adoption of draft mental health bill by the government of India, mental health is slowly gaining attention as a priority in India among the policymakers [1]. It is estimated that up to 40 % of HIV infected children are orphaned in India but little is known about their mental health consequences [2]. Mental health issues related to HIV/AIDS among young people, orphans and for those caring for orphans are well recognized globally, including depression, generalized anxiety, conduct and peer relationship problems, however, majority of the evidence comes from Africa [3–16]. Previous studies among Indian children have highlighted co-morbid conditions in children with depression to include anxiety and conversion/dissociative disorder [17], and the prevalence of anxiety disorder was reported to be 18 % in children infected with HIV [17]. We have recently reported the prevalence of depression to be 84.4 % among HIV orphaned children in Hyderabad from southern India [18].

In countries where local data are not available to help guide national policies to address the health issues of orphans and vulnerable children affected by HIV/AIDS, the UNAIDS recommends to replicate successful interventions that were implemented elsewhere [19]. With one or both parents dead for an estimated 5 % of the over 400 million children in India [20, 21], there is a strong need for mental health interventions targeting the orphans and vulnerable children irrespective of the cause of parental death. In this paper, we provide comparison of generalized anxiety, conduct and peer relationship problems among children orphaned by HIV/AIDS and those orphaned due to other disease/conditions to contribute to building local evidence to guide relevant policies and programs.

## Methods

We conducted a mental health study among orphaned children during January to March 2012 in 14 orphanages in and around Hyderabad city in southern India. The ethics approval for this study was provided by the Ethics Committee of the Public Health Foundation of India, New Delhi. Provision was made for referral to a psychologist if a child felt emotionally disturbed following the interview.

Detailed methodology for this study has been reported previously [18], and methods of relevance are presented here. We sampled children orphaned due to HIV/AIDS (COA) and those orphaned because of reasons other than HIV/AIDS (COO) aged 12 to 16 years. An orphan child was defined as a child who had lost one or both parents, and therefore included maternal, paternal, and double orphans [22]. A total of 14 orphanages having at least 20 orphaned children in the ages 12 to 16 years were sampled, and these together housed 524 orphaned children. Of these, two orphanages were run by the Government of the Indian state of Andhra Pradesh and the remaining 12 by private non-government organisations (NGOs). A total of 6 orphanages housed COO and 8 orphanages housed exclusively COA. Assuming 80 % power to detect a 10 % difference in mental health outcomes of interest between AIDS and other orphans at the 95 % confidence level (95 % CI 3.5–16.5 %), using the unpooled method we estimated a total sample size of 167 children from each among COA and COO. We utilized proportional sampling technique to maintain adequate representation of the COO to their estimated number available at each orphanage. However, we sampled all available eligible COA as the numbers of these children were not enough. The children aged 12–16 years who had spent at least 6 months at the orphanage and who could understand at least one of the three languages – Telugu, Hindi or English were considered eligible for the study.

Each potential participant was contacted by an interviewer trained in the study procedures with the assistance of the orphanage staff. The study was explained and informed consent sought for participation. For children aged 12 to 14 years, child assent and the consent from the concerned care-giver/guardian was obtained; and written informed consent was provided by children 15–16 years of age. All participants had the right to refuse participation or stop interview anytime. Before starting interview, each participant was narrated a short and simple story to assist the child to understand the context and content of the interview. This story was developed with inputs from a mental health expert with experience in dealing with children. After this narration, the interview was conducted in privacy. Average interview time was 45 min, and each participant received a nominal gift as a token of appreciation for their time at the end of interview.

The interview documented demographic characteristics of children including age, sex, education, religion, type of orphan, and duration of stay in orphanage. Mental health related measures documented relevant to this paper included generalized anxiety, history of abuse, violence and discrimination and conduct and peer relationship problems. History of abuse and violence by friends or relatives including type of abuse (denial of food, healthcare and other essential needs, denial of financial or property inheritance, physical and emotional abuse and sexual abuse), experience of abuse in the orphanage (denial of basic needs such as food and shelter, verbal abuse, threat of violence, physical beatings, sexual and mental abuse), and witnessing fights between parents was documented. History of experience of discrimination from friends/relatives and community were also documented.

We used the generalized anxiety domain of the Spence Children’s Anxiety Scale (SCAS) which is designed to assess general anxiety for children aged 8 to 15 years [23–25]. The respondents were asked to rate the degree to which they experienced each general anxiety symptom on a 4-point frequency scale (never, sometimes, often, and always). The possible scores ranged from 0 to 18, and score higher than 8 indicates elevated levels of generalized anxiety [23–25]. The Strengths and Difficulties Questionnaire (SDQ) which is designed to assess behavioural disorders in children aged 5 to 17 years was used to document conduct and peer relationship problems [26–30]. The respondents were asked to rate the degree to which they experienced each symptom on a 3-point frequency scale (not true, somewhat true, and certainly true). The possible scores ranged from 0 to 10. A score of 4 is considered borderline and that between 5 and 10 is considered abnormal for conduct problem; and a score of 4–5 is considered borderline and score between 6 and 10 is considered abnormal for peer relationship problem [26–30]. Both the scales were translated into the local languages for use, and then were back-translated and field-tested to ensure proper readability. As cultural validity was a major concern in translating this scale, the researchers closely collaborated with mental health experts, child counselors and the NGO/orphanage staff to achieve accuracy of cultural understanding and translation for these scales.

Four rounds of pre-testing of the entire study instrument were undertaken among orphaned children aged 12–16 years prior to the study by the study investigators in consultation with a psychologist who worked with children. Inputs from the mental health experts, child counselors and the NGO/orphanage staff were obtained to refine and validate these for the study population. Based on these exercises, certain definitions were simplified, revisions in local language translation for Hindi and Telugu were made, and interview techniques improved.

SPSS version 17.0 was used for data analysis. Descriptive statistics for generalized anxiety, and conduct and peer relationship problem scores are reported for relevant variables, and independent sample T test and ANOVA test were used to assess significance as appropriate [31]. We used the Tukey post-hoc test for independent variables with more than two categorical groups to assess which groups differed from each other [32]. The association of generalized anxiety score with depression, conduct and peer relationship problem score is presented separately for COA and COO; and these scores were compared using the bivariate regression types available in Microsoft Excel (linear). We report the prevalence of generalized anxiety score >8, conduct problem score >4, and peer relationship problem score >5 among these children, which are the clinical cut-off scores in western settings [23–30]. Multiple classification analysis (MCA) was performed to assess the variation in intensity of anxiety, and conduct and peer relationship problems with select factors including depression which we have previously reported [18]. 95 % confidence intervals (CI) are reported as appropriate. We used generalized anxiety, and conduct and peer relationship problem scores as continuous variable in MCA as clinical cut-off scores for these conditions are not readily available for young children in India. Among the items in SCAS and SDQ, we report the items which substantially contributed to the generalized anxiety, conduct and peer relationship problems in COA and COO groups, respectively.

## Results

A total of 400 orphaned children aged 12–16 years were approached from 14 orphanages of whom 396 (99.3 %) participated. Among those who participated, 199 (50.3 %) were COA and 306 (76.5 %) were aged 12 to 14 years and the median age for both boys and girls was 13 years. The proportion of boys was higher among the COA (63.5 %) than that of girls in the COO group (59 %). Paternal orphans constituted nearly half of all the children sampled (51.8 %) followed by double orphans (30.7 %). The average duration of stay in an orphanage was 3.2 years (range 0 to 8 years) for COA and 3.6 years (range 0 to 12 years) for COO.

### Distribution of scores

Table 1 shows the distribution of mean scores for generalized anxiety, conduct problem, and peer relationship problem with select variables for the orphaned children. The overall mean generalized anxiety, conduct and peer relationship problem scores were 11.1 (Standard Deviation, SD 5.2), 3.8 (SD 2.5) and 3.8 (SD 2.5) for COA and 7.6 (SD 4), 2.6 (SD 2) and 2.3 (SD 1.8) for COO, respectively. Among the COA group, the highest levels of mean generalized anxiety, conduct and peer relationship problem scores were observed among those children who had resided in an orphanage for >3 years and for girls. Among the COO, the highest levels of mean generalized anxiety, conduct and peer relationship problem scores were observed among those children who had reported experiencing abuse ever.

**Table 1:** Distribution of generalized anxiety (GA) score using the Spence Children’s Anxiety Scale [23–25] and of conduct (CP) and peer relationship problem (PRP) scores using the Strengths and Difficulty Questionnaire [26–30] by select variables among institutionalized orphaned children in Hyderabad

Variable	Categories	Children orphaned by HIV/AIDS (COA)							Children orphaned by other reasons (COO)						
		*N* = 199	Generalized anxiety score		Conduct problem score		Peer relationship problem score		*N* = 197	Generalized anxiety score		Conduct problem score		Peer relationship problem score	
			Mean	SD	Mean	SD	Mean	SD		Mean	SD	Mean	SD	Mean	SD
Age*	12 to 14 years	186	11.1	5.2	3.8	2.6	3.8	2.6	118	7.6	3.8	2.4	2	2.2	1.8
	15 to 16 years	13	11	5.5	4.2	1.6	3.9	2.3	79	7.6	4.1	2.9	1.9	2.5	2
Sex†	Boy	127	10.4	4.9	3.6	2.4	3.5	2.3	80	7.2	3.8	3.2	2.1	2.9	2.1
	Girl	72	12.3	5.5	4.2	2.7	4.3	2.8	117	7.9	4	2.2	1.7	1.9	1.6
Education‡	Never been to school/Class 1–5	131	10.7	5.3	3.7	2.6	3.9	2.6	44	8.6	4	2.9	2	3.3	2.2
	Class 6–12	68	11.7	5	4	2.4	3.7	2.4	153	7.3	3.8	2.5	2	2	1.6
Religion§	Hindu	60	10.2	5.7	3.6	2.3	3.1	2.6	140	7.1	3.8	2.6	1.9	2.2	1.8
	Non-Hindus	139	11.4	4.9	3.9	2.6	4.1	2.5	54	8.6	3.9	2.7	2.1	2.5	1.9
Duration of stay at the orphanage¶	<= 2 years	81	10.2	5.3	3.7	2.8	3.6	2.3	98	7.6	4.1	2.6	2.1	2.6	1.9
	3–4 years	75	12.4	4.8	4.2	2.5	4.6	2.7	36	7.8	3.3	2.6	1.7	2.1	1.6
	>4 years	43	10.4	5.3	3.4	2.1	2.8	2.2	63	7.4	4.1	2.6	1.9	2	1.9
Ever bullied or ill-treated by friend/relatives**	Yes	100	11.9	5.1	4.2	2.5	4.3	2.6	53	8.4	3.9	3.3	2.1	3.3	2.1
	No/Cannot say	99	10.2	5.2	3.4	2.5	3.3	2.4	144	7.3	3.9	2.4	1.9	1.9	1.6
Ever abused at orphanage††	Yes	38	12	4.5	4.1	2	3.7	2.4	46	8.5	3.3	3.7	2.1	3.2	1.9
	No	161	10.9	5.3	3.8	2.6	3.8	2.6	151	7.3	4	2.3	1.8	2	1.7
Ever witnessed fights between parents‡‡	Yes	100	12.1	5.4	2.1	0.8	1.9	0.8	71	8.6	3.7	1.8	0.9	1.4	0.6
	No/Don’t recall/Don’t know	99	10.1	4.9	1.7	0.9	1.7	0.8	126	7.1	4	1.4	0.7	1.2	0.5
Ever experienced discrimination§§	Yes	75	12.1	4.6	2.1	0.9	1.9	0.9	31	8.9	3.6	1.9	0.9	1.5	0.7
	No	124	10.5	5.5	1.8	0.9	1.7	0.8	166	7.4	3.9	1.4	0.7	1.2	0.5

SD refers to standard deviation

*Independent sample T test for significance: *p* = 0.846, 0.123 and 0.354 for GA, CP and PRP for COA; *p* = 0.437, 0.919, and 0.239 for GA, CP and PRP for COO

†Independent sample T test for significance: *p* = 0.437, 0.523 and 0.037 for GA, CP and PRP for COA; *p* = 0.880, 0.016 and 0.001 for GA, CP and PRP for COO

‡Independent sample T test for significance: *p* = 0.545, 0.235 and 0.426 for GA, CP and PRP for COA; *p* = 0.759, 0.780 and 0.016 for GA, CP and PRP for COO

§Independent sample T test for significance: *p* = 0.042, 0.320 and 0.534 for GA, CP and PRP for COA; *p* = 0.238, 0.955 and 0.640 for GA, CP and PRP for COO

¶ANOVA test for significance: *p* = 0.181, 0.343 and 0.004 for GA, CP and PRP for COA; Tukey post-hoc test for significance: p =0.024 for GA between 0 and 2 years and 3–4 years, *p* = 0.001 for PRP between 3 and 4 years and >4 years for COA; *p* = 0.044, 0.205 and 0.266 for GA, CP and PRP for COO

**Independent sample T test for significance: *p* = 0.611, 0.840 and 0.230 for GA, CP and PRP for COA; *p* = 0.982, 0.695 and 0.001 for GA, CP and PRP for COO

††Independent sample T test for significance: *p* = 0.066, 0.030 and 0.457 for GA, CP and PRP for COA; *p* = 0.093, 0.236 and 0.088 for GA, CP and PRP for COO

‡‡Independent sample T test for significance: *p* = 0.141, 0.203 and 0.436 for GA, CP and PRP for COA; *p* = 0.470, 0.338 and 0.088 for GA, CP and PRP for COO

§§Independent sample T test for significance: *p* = 0.018, 0.015 and 0.576 for GA, CP and PRP for COA; *p* = 0.631, 0.374 and 0.288 for GA, CP and PRP for COO

**Fig. 1:**
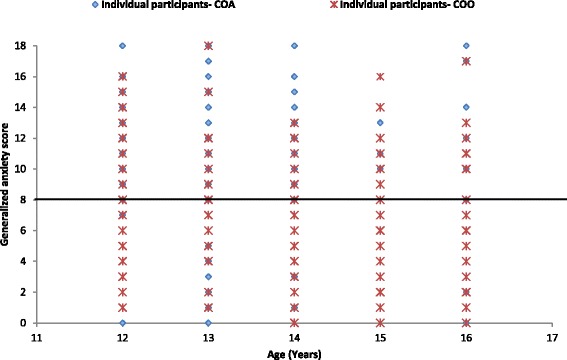
Distribution of generalized anxiety scores by age using the Spence Children’s Anxiety Scale [23–25] for the institutionalized orphaned children in Hyderabad. Horizontal line indicates the clinical cut-off score >8. COA denotes children orphaned by HIV/AIDS and COO denotes children orphaned by other reasons

**Fig. 2:**
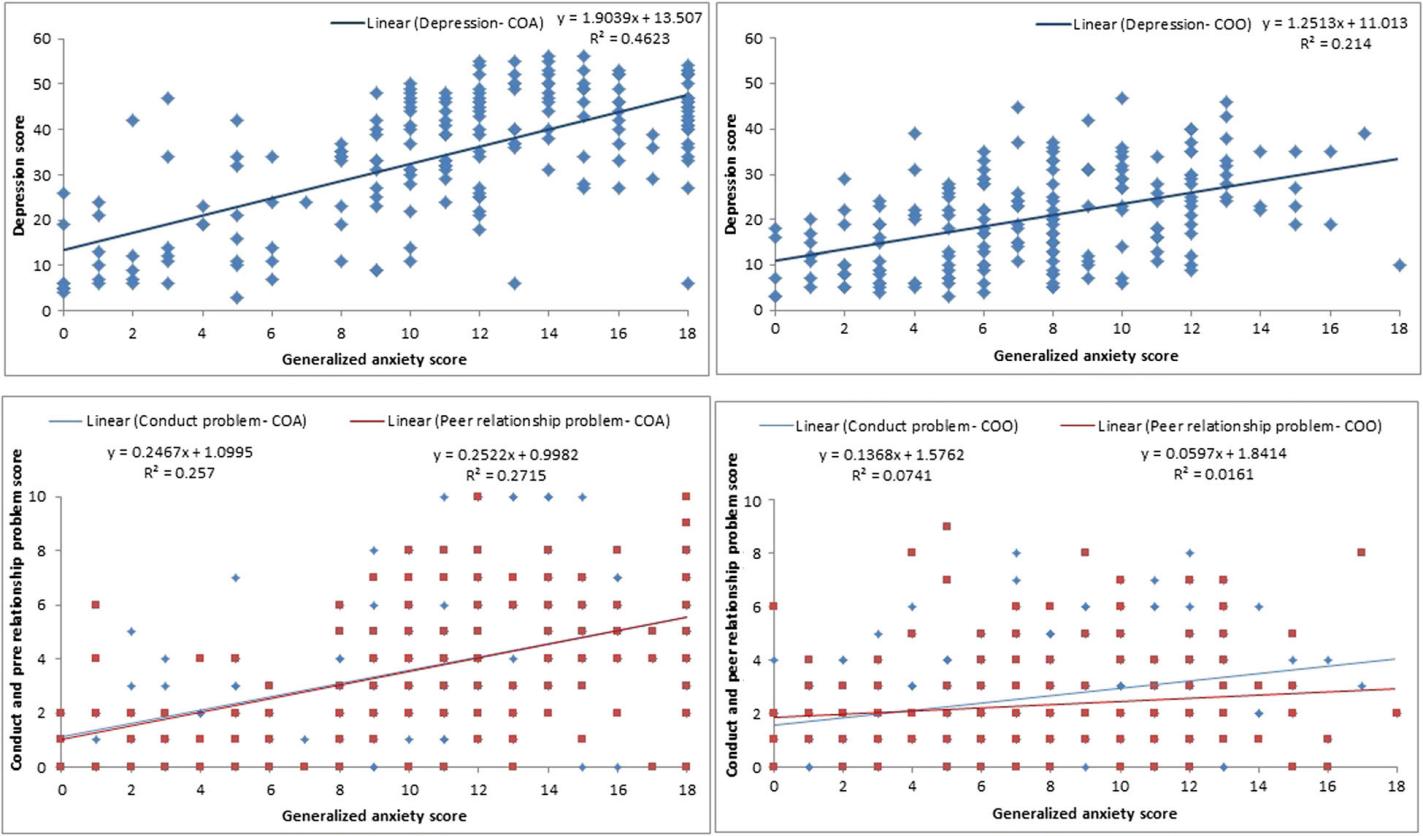
Distribution of generalized anxiety using the Spence Children’s Anxiety Scale [23–25] and depression, conduct and peer relationship problem scores using Strengths and Difficulties Questionnaire [26–30] for institutionalized orphaned children in Hyderabad. COA denotes children orphaned by HIV/AIDS and COO denotes children orphaned by other reasons

The distribution of generalized anxiety score for both COA and COO was clustered around the younger age groups (*p* = 0.001; Fig. 1). The association between generalized anxiety score with depression, conduct and peer relationship problem is shown in Fig. 2. Among both groups, generalized anxiety score increased with an increase in depression score and this association was much stronger for COA (*R*^2^ = 46.2 %) than for COO (*R*^2^ = 21.4 %). The levels of generalized anxiety and conduct and peer relationship problems were also positively associated for both the groups. However, in the COA group, generalized anxiety score increased similarly with increasing conduct (regression slope: 0.247) and peer relationship problem scores (regression slope: 0.252); whereas conduct problem registered a steep increase among the COO group (regression slope: 0.136) as compared to peer relationship problem (regression slope: 0.059) as the level of generalized anxiety increased. These two regression slopes were significantly different using pool error variance test (*p* < 0.001).

The overall prevalence of generalized anxiety score of > 8 was 56.1 % (95 % CI 51.1–60.9 %), and conduct problem score of >4 and peer relationship problem score of >5 was 25.3 % (95 % CI 21.2–29.8 %) and 17.2 % (95 % CI 13.8–21.2 %), respectively. The prevalence of generalized anxiety, conduct and peer relationship problem with these scores was significantly higher among COA–74.4 % (95 % CI 67.8–80.0 %; *p* < 0.001), 33.2 %, (95 % CI 26.9–40.1 %; *p* < 0.001) and 27.6 %, (95 % CI 21.8–34.3 %; *p* < 0.001), respectively. The prevalence of these in COO was 37.6 %, (95 % CI 31.0–44.6 %), 17.3 %, (95 % CI 12.6–23.2 %), and 6.6 %, (95 % CI 3.9–11.1 %), respectively.

### Characteristics of generalized anxiety

Table 2 shows distribution of items on the SCAS by the type of orphan. Overall, the items that were reported the most were: I felt afraid (always, 30.8 %), I worry about things (always, 29.8 %), and when I have a problem, my heart beats really fast (always, 25.8 %). COA were more likely to report “feeling afraid, worrying about things, and worrying that something bad would happen” as compared with the COO (*p* < 0.001).

**Table 2:** Distribution of Spence Children’s Anxiety Scale items [23–25] towards the burden of generalized anxiety among the orphaned children in Hyderabad (not mutually exclusive)

Item description	Total orphans (*N* = 396; % of N)				Children orphaned by HIV/AIDS (*N* = 199; % of N)				Children orphaned by other reasons (*n* = 197; % of N)			
	Never	Some-times	Often	Always	Never	Some-times	Often	Always	Never	Some-times	Often	Always
I worry about things	105	73	100	118	37	38	47	77	68	35	53	41
	(30.5)	(10.8)	(25.3)	(29.8)	(18.6)	(19.1)	(23.6)	(38.7)	(34.5)	(17.8)	(26.9)	(20.8)
When I have a problem, I get a funny feeling in my stomach	85	104	137	70	30	48	72	49	55	56	65	21
	(21.5)	(26.3)	(34.6)	(17.7)	(15.1)	(24.1)	(36.2)	(24.6)	(27.9)	(28.4)	(33.0)	(10.7)
I feel afraid	88	78	108	122	30	22	53	94	58	56	55	28
	(22.2)	(19.7)	(27.3)	(30.8)	(15.1)	(11.1)	(26.6)	(47.2)	(29.4)	(28.4)	(27.9)	(14.2)
When I have a problem, my heart beats really fast	46	100	148	102	16	47	60	76	30	53	88	26
	(11.6)	(25.3)	(37.4)	(25.8)	(8.0)	(23.6)	(30.2)	(38.2)	(15.2)	(26.9)	(44.7)	(13.2)
I worry that something bad will happen to me	141	90	76	89	56	34	32	77	85	56	44	12
	(35.6)	(22.7)	(19.2)	(22.5)	(28.1)	(17.1)	(16.1)	(38.7)	(43.1)	(28.4)	(22.3)	(6.1)
When I have a problem, I feel shaky	80	103	129	84	24	52	53	70	56	51	76	14
	(20.2)	(26.0)	(32.6)	(21.2)	(12.1)	(26.1)	(26.6)	(35.2)	(28.4)	(25.9)	(38.6)	(7.1)

Comparing boys and girls, boys were more likely to report that feeling afraid (always, 27.1 %; *p* = 0.002), feel that when in a problem, their heart beats really fast (always, 25.1 %; *p* = 0.070), and when in problem they feel shaky (always, 22.2 %; *p* = 0.173), whereas more girls likely to worry about things (always, 38.6 %; *p* = 0.002), and feeling afraid (always, 34.9 %; *p* = 0.002).

### Characteristics of conduct and peer relationship problem

Table 3 shows distribution of items on the SDQ for the study participants that measure conduct and peer relationship problems. The items that were reported the most for conduct problem were: I get very angry and often lose my temper (certainly true, 47.7 %) and I fight a lot (certainly true, 28 %). Both COA and COO reported “getting angry and often losing temper” followed by “I fight a lot” in the COA and “I usually do as I am told” in the COO group. For the peer relationship problem, the items that were reported the most were: I get on better with adults than with people of my age (certainly true, 46 %) followed by I am usually on my own (certainly true, 18.7 %). Both COA and COO were also more likely to report I get on better with adults than with people of my age (certainly true, 42.7 and 49.2 %) followed by I am usually on my own (certainly true, 27.6 and 9.6 %).

**Table 3:** Distribution of Strengths and Difficulties Questionnaire [26–30] items towards the burden of conduct and peer relationship problem among the orphaned children in Hyderabad (not mutually exclusive)

Item description	Total orphans (*N* = 396; % of N)			Children orphaned by HIV/AIDS (*N* = 199; % of N)			Children orphaned by other reasons (*N* = 197; % of N)		
	Not true	Somewhat true	Certainly true	Not true	Somewhat true	Certainly true	Not true	Somewhat true	Certainly true
Conduct problem									
I get very angry and often lose my temper	104	103	189	32	44	123	72	59	66
	(26.3)	(26.0)	(47.7)	(16.1)	(22.1)	(61.8)	(36.5)	(29.9)	(33.5)
I usually do as I am told	260	67	69	140	24	35	120	43	34
	(65.7)	(16.9)	(17.4)	(70.4)	(12.1)	(17.6)	(60.9)	(21.8)	(17.3)
I fight a lot	185	100	111	64	47	88	121	53	23
	(46.7)	(25.3)	(28.0)	(32.2)	(23.6)	(44.2)	(61.4)	(26.9)	(11.7)
I am often accused of lying or cheating	270	58	68	123	39	37	147	19	31
	(68.2)	(14.6)	(17.2)	(61.8)	(19.6)	(18.6)	(74.6)	(9.6)	(15.7)
I take things that are not mine	348	22	26	174	9	16	174	13	10
	(87.9)	(5.6)	(6.6)	(87.4)	(4.5)	(8.0)	(88.3)	(6.6)	(5.1)
Peer relationship problem									
I am usually on my own	253	69	74	99	45	55	154	24	19
	(63.9)	(17.4)	(18.7)	(49.7)	(22.6)	(27.6)	(78.2)	(12.2)	(9.6)
I have one good friend or more	278	87	31	109	66	24	169	21	7
	(70.2)	(22.0)	(7.8)	(54.8)	(33.2)	(12.1)	(85.8)	(10.7)	(3.6)
Other people of my age generally like me	248	113	35	100	77	22	148	36	13
	(62.6)	(28.5)	(8.8)	(50.3)	(38.7)	(11.1)	(75.1)	(18.3)	(6.6)
Other children or young people tease or harass me	266	77	53	103	57	39	163	20	14
	(67.2)	(19.4)	(13.4)	(51.8)	(28.6)	(19.6)	(82.7)	(10.2)	(7.1)
I get on better with adults than with people of my age	101	113	182	52	62	85	49	51	97
	(25.5)	(28.5)	(46.0)	(26.1)	(31.2)	(42.7)	(24.9)	(25.9)	(49.2)

### Determinants of generalized anxiety, conduct and peer relationship problems

Table 4 shows the MCA for the adjusted predicted mean scores for generalized anxiety, conduct and peer relationship problem scores. As expected, depression had the highest effect on the intensity of generalized anxiety, conduct and peer relationship problems with a beta value of 0.477, 0.379 and 0.453, respectively. Being an AIDS orphan and being a girl had the most impact on generalized anxiety (0.100 and 0.115, respectively) as compared with their effect on conduct or peer relationship problems. Having witnessed their parents fight had a relatively higher impact on the conduct problem (0.142) whereas duration of stay in orphanage and ever being bullied had the most impact on the peer relationship problems (0.153 and 0.134) respectively.

**Table 4:** Multiple classification analysis for effect of selected variables on generalized anxiety, conduct and peer relationship problem for the institutionalized orphaned children in Hyderabad

Variable	Categories	*N* = 396	Adjusted predicted Mean								
			Generalized anxiety			Conduct problem^a^			Peer relationship problem^b^		
			Mean	Beta	P value	Mean	Beta	P value	Mean	Beta	P value
Age	12 to 14 years	304	9.55	0.077	0.086	3.21	0.010	0.836	3.11	0.044	0.324
	15 to 16 years	92	8.65			3.26			2.87		
Sex	Boy	207	8.80	0.115	0.005	3.35	0.057	0.196	3.13	0.033	0.423
	Girl	189	9.93			3.08			2.97		
Child orphaned by HIV/AIDS	Yes	199	9.83	0.100	0.047	3.30	0.034	0.530	3.14	0.037	0.462
	No	197	8.85			3.14			2.97		
Duration of stay at the orphanage	≤2 years	179	9.00	0.103	0.047	3.12	0.076	0.225	3.08	0.153	0.001
	3 to 4 years	111	10.15			3.51			3.50		
	>4 years	106	9.07			3.10			2.54		
Witnessed fights between parents	Yes	171	9.99	0.116	0.005	3.60	0.142	0.001	3.23	0.066	0.111
	No/do not remember	225	8.85			2.93			2.92		
Ever bullied or ill-treated by friend/relatives	Yes	153	9.53	0.031	0.530	3.45	0.078	0.144	3.45	0.134	0.007
	No/ Cannot say	243	9.22			3.08			2.80		
Ever experienced discrimination	Yes	106	9.57	0.028	0.571	3.28	0.014	0.795	3.09	0.009	0.849
	No	290	9.26			3.20			3.04		
Depression score^c^	<= mean score	199	7.01	0.477	<0.001	2.34	0.379	<0.001	2.00	0.453	<0.001
	> mean score	197	11.70			4.11			4.12		
	Full model	396		0.379	<0.001		0.252	<0.001		0.354	<0.001

^a^The conduct problem scale from the Strengths and Difficulties Questionnaire was used to assess conduct problem in children. The possible scores ranged from 0 to 10, and a score of 4was considered borderline and scores 5–10 are considered abnormal for conduct problem

^b^The peer relationship problems scale from the Strengths and Difficulties Questionnaire was used to assess peer relationship problems in children. The possible score ranged from 0 to 10, and a scores of 4–5 are considered borderline and scores 6–10 are considered abnormal for peer relationship problem

^c^The Center for Epidemiologic Studies-Depression scale (CES-DC) designed for children aged 6 to 17 years was used to measure depression. The possible scores ranged from 0 to 60, and A CES‐DC score of 15 or higher has previously been considered suggestive of significant level of depressive symptoms in children and adolescents

## Discussion

We found a significantly higher proportion of AIDS orphans dealing with generalized anxiety, conduct and peer relationship problem as compared with orphans due to other reasons in the institutionalized orphans in the city of Hyderabad.

A little over half of the children in this study had a generalized anxiety score of >8, and 75 % of COA and 38 % of COO were identified with this score. Generalized anxiety is often underestimated as it remains undiagnosed owing to the internalized nature of its symptoms [7], and is associated with substantial negative effects on social, emotional and academic success of those affected [8]. There are mixed reports from previous research on anxiety in COA with some studies reporting a high level of anxiety [4, 9–12], and some lower levels [13, 33]. The mean generalized anxiety scores in our study were higher among girls, particularly COA, than boys. Furthermore, girls were more likely to report being worried about things and feeling afraid, which is consistent with previous research which emphasizes that factors like personal, physical, biological, socio-cultural, and coping mechanisms could influence anxiety levels among girls [34–36]. These findings point to the need for more research to understand the dynamics of gender and its role in influencing mental health outcomes among children and adolescents in India. In the meanwhile, these findings can be used to develop gender-specific social support programs that address the emotional needs of orphans.

The levels of generalized anxiety and depression revealed a positive association in both the groups, with COA exhibiting a strong positive correlation. Although depression and anxiety have historically been seen as distinct conditions, the two disorders are not mutually exclusive and often coexist to varying degrees in the same individual [37, 38], and patients with co-morbid depression and anxiety frequently also have poorer prognosis and a lower response to treatment [37, 38]. This study finding has a strong intervention implication for mental health outcomes among orphans as the strategies aiming to improve child mental health under the current child mental health policy and school mental health policy should aim to look into the interplay between anxiety-depression and design appropriate interventions [1].

In this study, the mean scores for conduct and peer relationship problem were significantly higher among those who were bullied or ill-treated by their friends or relatives and those who experienced discrimination as an orphan. This was more explicitly visible in the COA than the COO, with a significantly higher proportion of COA identified with conduct and peer relationship problem scores >4 and >5, respectively. The relationship between parental death due to AIDS on children’s conduct and peer relationship problems is well documented [39–41]. The possibility of psychopathic behavior among children raised without supervision relating to unknown psychosocial effects of orphanhood has been noted previously [11, 42]. In this study, the orphaned children reported getting very angry often and fighting frequently; and most of the COA reported having just one or no friends at all and expressed concerns that their peers generally do not like them. The majority also noted that they got along well with older people rather than their peers. These finding support previous and growing evidence which suggest that orphanhood by AIDS is significantly associated with increased peer relationship problems [34, 43]. It is reasoned that it is cumulative effects of HIV/AIDS related stigma and discriminations that underline the higher scores of peer relationship problems in COA over COO [44]. The main implication of these findings is the need to recognize and address the increased risk of conduct and peer relationship problem disturbances in these children by the government agencies, NGO’s, and by health professionals working with HIV/AIDS affected children.

The strength of this study is that this is the largest study to date that has compared psychological issues among COA and COO living in orphanages. With twice the prevalence of generalized anxiety and conduct problems, and four times higher prevalence of peer relationship problems among the COA than COO, it is clear that a parental death due to HIV/AIDS has far reaching mental health implications than parental death due to any other reason. There are some limitations of this study to be taken into consideration. As validated mental health scales for children are not readily available in India, we used SCAS which has been used in various cultures. However, all psychological measures should be interpreted with caution in different cultures. We, therefore, used continuous scale and not the clinical cut-off score reflecting western norms as it may be inappropriate for this study population. Misreporting of the parental cause of death could be a limitation as it was documented based on information provided by the child or the NGO staff. It was not possible to confirm the parental cause of death. The cross-sectional nature of these data does not allow temporal or causal explanations as these data do not allow comment on psychological issues in these children prior to them being orphaned.

Psychosocial needs of children affected by AIDS, especially orphans, are most often neglected in the program design. There is a lack of specific program or policy in India to address the growing numbers of COA [45, 46]. Already four years into the phase IV of the national AIDS control program, interventions for children orphaned by AIDS are conspicuous by their absence [47]. There is clearly a need for specific interventions based on local evidence to have an effective response for these vulnerable groups through the national AIDS program, and as part of the child mental health policy, school mental health policy, and mental health policies for disabled at the community level [1].

## Conclusions

In conclusion, this study has contributed to building an evidence and further work is neededto understand long-term impacts of parental death on children. The urgent need to address the poor mental health of orphans, in particular the AIDS orphans, in India is highlighted.
